# Willingness of Lebanese physicians in the United States to relocate to Lebanon

**DOI:** 10.1186/1478-4491-10-15

**Published:** 2012-07-10

**Authors:** Elie A Akl, Nancy Maroun, Khalil El-Asmar, Eliane Abou Jaoude, Jihad Irani, Kamal Badr

**Affiliations:** 1 Department of Medicine, University at Buffalo, ECMC-DKM 216, 462 Grider St, Buffalo, NY, 14215, USA; 2 Department of Family Medicine, University at Buffalo, Buffalo, NY, USA; 3 Department of Clinical Epidemiology and Biostatistics, Hamilton, ON, Canada; 4 Department of Sociology, Buffalo State College, Buffalo, NY, USA; 5 Department of Epidemiology and Population Health, American University of Beirut, Beirut, Lebanon; 6 School of Medicine and Biomedical Sciences, University at Buffalo, Buffalo, NY, USA; 7 Faculty of Medicine and Medical Sciences, University of Balamand, Beirut, Lebanon; 8 Department of Internal Medicine, American University of Beirut, Beirut, Lebanon

**Keywords:** Physician migration, Repatriation, Lebanese physician workforce

## Abstract

**Background:**

We recently proposed that Lebanon could become a regional ‘academic hub’ through the repatriation of emigrated Lebanese physicians who would then provide clinical services in the Arab Gulf region on a locum tenens basis. The objectives of this study were to assess the willingness of Lebanese medical graduates practicing in the United States of America to relocate to Lebanon and the Arab Gulf region and to explore the factors associated with this willingness.

**Methods:**

In 2009 we surveyed Lebanese medical graduates practicing medicine in the United States. The questionnaire included questions about their willingness to relocate to Lebanon and to the Arab Gulf region and the associated timeframes. The questionnaire also included questions about family factors. We linked responders’ answers to their personal, educational, and practice characteristics provided by the American Medical Association Physicians’ Dataset. We conducted both descriptive and regression analyses.

**Results:**

A total of 286 physicians participated in the survey (57% response rate). A majority (61%) was willing to relocate to Lebanon (51% possibly, 10% definitely). A third (33%) were willing to relocate to the Arab Gulf region (31% possibly, 2% definitely). About half (54%) were willing to relocate to Lebanon as a base for clinical missions to the Arab Gulf region (49% possibly, 5% definitely). Willingness to relocate to Lebanon was independently associated with Lebanese citizenship and the birthplace of the spouse being Lebanon, and inversely associated with US citizenship. Willingness to relocate to the Arab Gulf region was independently associated with being board certified, and inversely associated with being married, the age of the oldest child, and practicing in direct patient care. Willingness to relocate to Lebanon as a base was not independently associated with any factor.

**Conclusions:**

The findings of this study support the feasibility of the proposal of Lebanon becoming a regional ‘academic hub’. Future research should explore other factors important for the feasibility of the proposal as well as actual relocation.

## Background

We recently proposed that Lebanon could become a regional ‘academic hub’ [1]. Under this proposal, Lebanese academic medical institutions would establish collaborative efforts with regional medical centres. They would also build a pool of high-quality human resources that would serve as a resource for the region [1]. Under one of the suggested models, repatriated Lebanese physicians (and their families) would be based in Lebanon while providing clinical services in the Arab Gulf region on a locum tenens basis (i.e., filling a job position on a temporary basis) [1], or through Lebanon-based regional health care delivery systems.

The primary objective of this survey was to assess the willingness of Lebanese medical graduates (LMGs) practicing in the United States to relocate to Lebanon and the Arab Gulf region. The secondary objective was to explore the factors associated with this willingness.

## Main text

### Methods

The target population of this mail survey consisted of LMGs practicing medicine in the United States. We excluded physicians in training. The sampling frame consisted of LMGs included in the 2009 American Medical Association Physicians Dataset (AMA-PD). The institutional review board of the State University of New York approved the study.

The survey questionnaire [See Additional file 1] was in English and included questions about family characteristics, and the willingness to relocate to: (1) Lebanon; (2) the Arab Gulf region; and (3) Lebanon with locum tenens missions to the Arab Gulf region. The three answer options to the three willingness questions were: ‘definitely not’, ‘possibly yes’, and ‘definitely yes’. Respondents reporting a willingness to relocate also specified in what number of years they expect to actually relocate. We also used additional demographic, educational, and practice data included in the AMA-PD.

We conducted descriptive analyses and ran regression models in which the dependent variable was the willingness question (reference category: ‘definitely not’) and the independent variables were the demographic, educational, practice and family characteristics.

## Results

Of a sample of 500 physicians we attempted to contact, 286 participated in the survey (57% response rate; see Additional file 2).

A majority (61%) was willing to relocate to Lebanon (51% possibly, 10% definitely). Table 1 shows that the estimated number of years prior to relocation was longer for those who expressed possible (compared to definite) willingness to relocate. In a regression analysis including all subjects, possible willingness to relocate was associated with Lebanese citizenship (odds ratio (OR) = 3.20; 95% confidence interval (CI) 1.71-5.96) while definite willingness to relocate was associated with Lebanese citizenship (OR = 6.73; 95% CI 2.04-22.22) and inversely associated with United States citizenship (OR = 0.30; 95% CI 0.09-0.94). In a regression analysis excluding single or engaged subjects, possible willingness to relocate was associated with Lebanese citizenship (OR = 2.84; 95% CI 1.32-6.13) and the birthplace of spouse being Lebanon (OR = 2.17; 95% CI 1.32-3.57) while definite willingness to relocate was not associated with any factor.

**Table 1:** Willingness to relocate to Lebanon and the Arab Gulf region of survey respondents (n = 286)

**Willingness to relocate to:**		**n; percentage (95% Confidence Interval)**	**Number of years to relocate (*Mean (sd)*)**
Lebanon	Definitely not	105; 37% (31%-42%)	Not applicable
	Possibly yes	147; 51% (46%-57%)	9.9 (5.3)^a^
	Definitely yes	29; 10% (7%-14%)	4.2 (4.2)^b^
The Arab Gulf region^c^	Definitely not	190; 66% (61%-72%)	Not applicable
	Possibly yes	88; 31% (25%-36%)	6.7 (4.1)^d^
	Definitely yes	6; 2% (1%-4%)	3.3 (1.9)^e^
Lebanon as a base for clinical missions to the Arab Gulf region^g^	Definitely not	128; 45% (39%-51%)	Not applicable
	Possibly yes	141; 49% (44%-55%)	6.3 (3.9)^f^
	Definitely yes	14; 5% (2%-7%)	3.1 (4.5)^h^

^a^ Data missing for n = 16.

^b^ Data missing for n = 2.

^c^ Data missing for n = 2.

^d^ Data missing for n = 15.

^e^ Data missing for n = 0.

^f^ Data missing for n = 22.

^g^ Data missing for n = 3.

^h^ Data missing for n = 4.

A third (33%) were willing to relocate to the Arab Gulf region (31% possibly, 2% definitely). Table 1 shows that the estimated number of years prior to relocation was longer for those who expressed possible (compared to definite) willingness to relocate. In a regression including all subjects, possible willingness to relocate was associated with being board certified (OR = 4.00; 95% CI 1.90-8.39), and inversely associated with being married (OR = 0.56; 95% CI 0.36-0.87), and practicing in direct patient care (OR = 0.92; 95% CI 0.86-0.99). Definite willingness to relocate was not associated with any factor. In a regression analysis excluding single or engaged subjects, possible willingness to relocate was associated with being board certified (OR = 3.10; 95% CI 1.25-7.48), and inversely associated with the age of oldest child (OR = 0.95; 95% CI 0.91-0.99). Definite willingness to relocate was not associated with any factor.

About half (54%) were willing to relocate to Lebanon as a base for clinical missions to the Arab Gulf region (49% possibly, 5% definitely). Table 1 shows that the estimated number of years prior to relocation was longer for those who expressed possible (compared to definite) willingness to relocate. In regression analyses, willingness to relocate was not associated with any factor.

## Discussion

The main limitation of this study is that we studied the willingness to relocate while there is no evidence that such willingness is predictive of actual relocation. Also, we were not able to identify a conceptual framework to support the development of the survey questionnaire.

The percentage of LMGs definitely not willing to relocate to Lebanon (37%) is significantly higher than the 10% of Lebanese medical students planning to train abroad (mostly in the United States) but never to return to Lebanon [2]. While this difference could be due to a generational effect, it may be related to physicians changing their plans by the time they start practicing in the United States.

A 2002 survey among international medical graduates in the United Kingdom found that 17% of those who graduated from high-income countries versus 50% of those who graduated from low-income countries intended to return [3]. A 2005 study evaluated the actual return ‘home’ of international medical graduates who at one point were licensed in Canada. While overall, 43% returned home, the percentage varied from 6.7% for graduates of Eastern European countries to 88.5% for graduates of Saudi Arabia [4]. These results demonstrate that return patterns are specific to both the source and destination countries.

The association of willingness to relocate to Lebanon with Lebanese citizenship and the birthplace of the spouse suggest the importance of family factors in making the relocation decision. While more research is needed to define these factors, it has been suggested – although not empirically studied - that the motives of Pakistani medical graduates for returning home include the aging of the parents, family ties, and a desire to raise children in a familiar culture [5].

The inverse association between willingness to relocate to Lebanon and having United States citizenship might be confounded by the time since migration; those who have migrated for a longer time are more likely to be documented and less likely to go back as they become integrated.

Our findings suggest that the proposal of making Lebanon a regional ‘academic hub’ by recruiting LMGs practicing in the United States is feasible. The percentage of those willing to relocate to Lebanon as a base for a job in the Arab Gulf region (54%) was higher than the percentage of those willing to relocate directly to that region (33%). However, the findings suggest a challenge to the proposal of making Lebanon a regional ‘academic hub’, namely the number of academicians who would be willing to relocate. In fact, LMGs employed by United States medical schools (representing the pool of academicians) constitute only 5% of all LMGs in the US.

## Conclusions

Lebanese medical schools should create adequate environments and incentives to attract migrant LMGs and retain them. They also need to explore the cost implications and business models needed for the success of this plan. Also, and in order to better target the repatriation efforts, there is a need to assess and monitor the exact and specific need for clinicians, physician educators and physician scientists.

Future research should study actual relocation of LMGs. There is also a need to qualitatively study the reasons for the relatively low percentage of LMGs definitely willing to relocate to Lebanon.

## Abbreviations

LMGs, Lebanese medical graduates; AMA-PD, American Medical Association Physicians’ Dataset.

## Competing interests

The authors declare that they have no competing interests.

## Authors’ contributions

EAA: study conception and design, data collection, data analysis, data interpretation, article drafting, obtaining of funding; NM: study conception and design, data collection, data interpretation; KEA: data analysis, data interpretation; EAJ: data collection; JI: data collection; KB: study design, data interpretation, obtaining of funding; All authors critically revised the article and provided final approval.

## Supplementary Material

Additional file 1 **Survey questionnaire.** Provides the survey questionnaire. Click here for file

Additional file 2 **Demographic, family, educational and practice characteristics of survey respondents.** Reports the demographic, family, educational and practice characteristics of survey respondents. Click here for file
