# Supplementary material for: Willingness of Lebanese physicians in the United States to relocate to Lebanon
Source: Hum Resour Health. 2012 Jul 10;10:15. doi: 10.1186/1478-4491-10-15 (PMC3549762; doi:10.1186/1478-4491-10-15)
Supplement: Additional file 1 — Survey questionnaire. Provides the survey questionnaire. [file 1478-4491-10-15-S1.doc]

1. **Citizenship** (check all that apply)

⁯ Lebanese ⁯ US citizenship ⁯ US green card ⁯ Other: ____________

1. **Marital Status**

⁯ Single ⁯ Engaged ⁯ Married ⁯ Divorced ⁯ Widow

1. **Place of birth of your spouse or fiancé(e)**

⁯ Lebanon ⁯ USA ⁯ Other:____________ ⁯ Not applicable

1. **Number of children**: _______
2. **Age of oldest child**: _______ year(s) ⁯ Not applicable
3. **Is at least one of your spouse parents or your parents settled in Lebanon?**

⁯ Yes ⁯ No

1. **Is at least one of your spouse parents or your parents settled in the US?**

⁯ Yes ⁯ No

1. **Willingness to relocate to Lebanon:**

⁯ Definitely not

⁯ Possibly yes; in about _____ years

⁯ Definitely yes; in about _____ years

1. **Willingness to relocate to the Arab Gulf region**

⁯ Definitely not

⁯ Possibly yes; in about _____ years

⁯ Definitely yes; in about _____ years

1. **Willingness to relocate to Lebanon as a base for clinical missions to the Arab Gulf region, for a few weeks at a time (locum tenens):**

⁯ Definitely not

⁯ Possibly yes; in about _____ years

⁯ Definitely yes; in about _____ years
