# Supplementary material for: Willingness of Lebanese physicians in the United States to relocate to Lebanon
Source: Hum Resour Health. 2012 Jul 10;10:15. doi: 10.1186/1478-4491-10-15 (PMC3549762; doi:10.1186/1478-4491-10-15)
Supplement: Additional file 2 — Demographic, family, educational and practice characteristics of survey respondents. Reports the demographic, family, educational and practice characteristics of survey respondents. [file 1478-4491-10-15-S2.doc]

**Additional file 2:** Demographic, family, educational and practice characteristics of survey respondents (n=286)

|  |  | *Missing data (n)* | *Mean (sd)* |
| --- | --- | --- | --- |
| Age |  | 3 | 48.4 (11.5) |
| Number of children |  | 46 | 2.1 (1.2) |
| Years since graduation |  | 3 | 24.0 (11.2) |
|  |  |  | *%* |
| Gender | Female | 3 | 49 (17%) |
| Country of Birth | Lebanon  Syria  Other | 125 | 129 (45%)  11 (4%)  10 (4%) |
| Citizenship^*^ | Lebanese citizenship  US citizenship  US Green Card  Other | 0 | 212 (74%)  194 (68%)  48 (17%)  18 (6%) |
| Marital status | Married  Single  Divorced  Widowed | 1 | 237 (83%)  33 (12%)  12 (4%)  3 (1%) |
| Country of birth of spouse or fiancé(e) | Lebanon  USA  Other  Not applicable | 0 | 168 (59%)  45 (16%)  39 (14%)  34 (12%) |
| At least one of spouse parents or own parents settled in Lebanon |  | 0 | 205 (72%) |
| At least one of spouse parents or own parents settled in the US |  | 0 | 115 (40%) |
| Medical School | American University of Beirut  St Joseph’s University  Lebanese University  Beirut Arab University | 3 | 207 (72%)  56 (20%)  19 (7%)  1 (<1%) |
| Board certification | Yes | 66 | 220 (77%) |
| US geographic area | Northeast  Midwest  South West | 4 | 71 (25)  82 (29)  96 (34)  33 (12) |
| Professional Employment | Group Practice  Self-employed solo practice  Government Facility  Medical School  Other  No Classification | 3 | 93 (33%)  58 (20%)  29 (10%)  15 (5%)  26 (9%)  62 (22%) |
| Primary Specialty | Internal Medicine  General Surgery  Pediatrics  Obstetrics and Gynecology  Family Medicine  Other | 3 | 29 (10%)  17 (6%)  14 (5%)  14 (5%)  9 (3%)  200 (70 %) |
| Type of Practice | Direct patient care  Unspecified  Medical Research  Medical Teaching  Administration | 3 | 247 (86%)  28 (10%)  4 (1%)  3 (1%)  1 (0%) |

^*^ Of 212 Lebanese citizens, 136 (54%) had US citizenship and 34 (16%) had permanent residency
